# Team Survivors: Preliminary Analysis of an Innovative Intervention to Promote Physical Activity in Survivors of Childhood Cancer and Hematopoietic Stem Cell Transplant

**DOI:** 10.3390/children12040399

**Published:** 2025-03-21

**Authors:** Meghan K. Flannery, Jocelyn Morin, Katrina O’Malley, Debra Schmidt, Jennifer A. Hoag

**Affiliations:** 1 Nationwide Children’s Hospital, Columbus, OH 43205, USA; mf7946a@american.edu; 2 Children’s Wisconsin, Milwaukee, WI 53226, USA; jmorin@childrenswi.org (J.M.); dschmidt@childrenswi.org (D.S.); 3 Department of Pediatrics, Medical College of Wisconsin, Milwaukee, WI 53226, USA

**Keywords:** survivorship, physical activity, cancer, health related quality of life, hematopoietic stem cell transplant

## Abstract

(1) Background: Inadequate physical activity is an ongoing issue for pediatric survivors of childhood cancer and hematopoietic stem cell transplant (HSCT), increasing their risk for chronic health conditions and decreasing quality of life. Team Survivors is a 12-week program in which survivors train as a group to participate in a community triathlon. Preliminary findings from a quality improvement project of Team Survivors were used to assess its feasibility in influencing survivors’ physical activity and quality of life and their family’s perception of the program. (2) Methods: Repeated measures *t*-tests evaluated changes in caregiver-reported exercise self-efficacy and health-related quality of life (HRQoL). These findings were supplemented by a narrative review of qualitative data from caregivers and survivors on their overall experience and satisfaction with the program. (3) Results: All participants successfully completed the triathlon, and families reported satisfaction with the program’s logistics (i.e., coordination, coaching, and practice). Neither caregiver-reported exercise self-efficacy nor HRQoL significantly differed between pre- and post-intervention, but notable improvements were reported in all domains of HRQoL. Qualitatively, the perceived benefits reported by caregivers and survivors were multifaceted. Physical benefits included improvements in survivors’ physical activity level and endurance. Psychosocially, caregivers and survivors reported greater confidence and motivation for physical activity, improved coping, and increased social engagement and feelings of normalcy. (4) Conclusions: Mixed method results support the feasibility of Team Survivors in positively impacting survivors’ ability to engage in physical activity. The multifaceted structure of the program may more broadly impact psychosocial functioning in addition to physical activity. Future studies require a larger sample size to adequately power the analyses.

## 1. Introduction

After the completion of hematopoietic stem cell transplant (HSCT) and cancer treatment, childhood survivors and their families are faced with managing the immediate and long-term consequences of treatment. Survivors are at a significantly heightened risk of developing a range of debilitating health complications due to treatment. The St. Jude Lifetime Cohort Study found that 98.2% of childhood cancer survivors had at least one chronic health condition (CHC) by their early 30s; 67.7% had a serious, disabling, or life-threatening CHC [1]. The gap between healthy controls and childhood cancer survivors exponentially increases with age, with childhood survivors having an average of 17 CHCs by age 50, while same-aged, healthy controls had only 9 [2]. Similarly, childhood survivors experience deficits in psychosocial functioning and overall health-related quality of life (HRQoL) both immediately and decades after treatment [3]. At the end of treatment, survivors often report social isolation, emotional distress, posttraumatic stress symptoms, fear of relapse, identity confusion, and the loss of supportive relationships with treatment teams [4,5]. 

Research has repeatedly found that less than 50% of survivors meet the exercise guideline- of an average of 60 min/day of moderate-to-vigorous intensity physical activity established by the World Health Organization [6,7,8,9]. Achieving this level of physical activity is strongly associated with improved physical (e.g., cardiometabolic health), mental (e.g., reduced depression), and cognitive (e.g., higher academic performance) health outcomes [9]. Conversely, increased sedentary behavior is associated with detrimental health outcomes, such as increased adiposity and fewer prosocial behaviors [9]. Impaired physical activity in childhood survivors—initially due to the deconditioning effects of treatment—is often maintained long into remission and adulthood. The deficits in physical activity for childhood survivors are especially concerning given their already elevated risk for serious health issues and reduced quality of life. Mounting efforts have been directed at improving physical activity in childhood survivors to ameliorate the adverse consequences of treatment. Childhood survivors who engage in regular physical activity have significantly higher quality of life, improved cardiovascular health, and decreased risk of all-cause mortality than those who do not [7,10,11]. In a study exploring exercise intolerance in adult survivors of childhood cancer, survivors who engaged in at least 150 min of moderate-to-vigorous physical activity a week were found to have significantly better cardiac functioning, as measured by ventricular ejection fraction and global longitudinal strain, and they had lower odds of experiencing exercise intolerance, which is associated with heart failure and death, than those who did not [12]. Pediatric survivors have also been found to have more risk factors for cardiovascular disease, such as greater android fat mass and lower systolic blood pressure, than a control group [13]. However, these risk factors were more greatly associated with physical activity and fitness in survivors than in the control group, suggesting increased physical activity and fitness might play a larger role in cardiovascular health for survivors of childhood cancer than their healthy peers.

Despite extensive evidence establishing the positive outcomes of regular physical activity, exercise programs designed for childhood cancer and HSCT survivors have produced mixed results [14,15,16]. Several studies have identified individual, psychological, environmental, social, and health-related factors influencing the physical activity of pediatric survivors [17,18,19]. Gilliam and Schwebel [20] proposed a socio-ecological framework conceptual model of childhood survivors’ physical activity that identifies the multi-level influences of medical, social/cultural, environmental, cognitive/emotional, and behavioral factors. This model emphasizes the importance of targeting both behavioral and social factors to effectively increase survivors’ physical activity. The need for a multifaceted intervention for physical activity may be especially prudent for childhood survivors given the intrinsic relationship between the development of physical and socioemotional skills at their age level. The majority of childhood physical activity occurs within social settings such as schools (e.g., structured physical education, walking between classes, recess), organized sports, and social interactions with friends [21,22,23]. Children are less likely than adults to engage in individual exercise at home or in a gym [24]. Family and peer support of physical activity and survivor- and caregiver-reported exercise self-efficacy are other unique predictors of survivors’ level of physical activity [25,26,27].

Given the direct and indirect cross-effects between physical and psychosocial functioning, an intervention to improve survivors’ physical activity may simultaneously enhance their psychosocial functioning [28,29,30]. The positive effects of physical activity on psychosocial outcomes are well-established [31,32]. Both structured (e.g., physical education in school and organized sports) and unstructured forms of physical activity (e.g., riding bikes and playing at parks with friends) provide core experiences for developing social skills, prosocial behaviors, and meaningful peer relationships in early and middle childhood [33,34,35]. A series of multilevel meta-analyses found physical activity interventions in adolescence have small-to-moderate effects on reduced externalizing and internalizing symptoms while improving self-concept and academic achievement [36]. Further, group exercise has been repeatedly shown to improve mental health, confidence, and social connectedness [37,38,39]. Specifically for cancer survivors, group exercise programs can enhance their sense of control over their cancer, body, and emotions. They can also help them regain a sense of normalcy, nurture identity exploration, increase self-efficacy, and promote social connection and support [10,40,41].

This study presents preliminary findings on the impact of Team Survivors on survivors’ physical and psychosocial functioning, using data from an ongoing quality improvement project. Team Survivors is a triathlon training program for pediatric survivors of childhood cancer and HSCT aimed at building community, increasing strength and flexibility, and creating lifelong physical activity habits for pediatric survivors. The program launched in 2019 as a collaboration between the cancer survivorship program and the sports medicine department at Children’s Wisconsin. Four cohorts (2019, 2022, 2023, and 2024) have completed the program since its inauguration. Unlike most exercise interventions for survivors, Team Survivors prioritizes improving survivors’ self-efficacy and social connectedness with other survivors and noncancer community members through physical activity. The multifaceted approach of Team Survivors addresses several identified barriers to physical activity for childhood survivors, which may bolster its effectiveness and promote engagement in physical activity that is maintained after the program’s completion.

## 2. Materials and Methods

### 2.1. Study Design, Procedures, and Data Collection

This study utilizes the first wave of data from the Team Survivors quality improvement project and includes pre- and post-intervention data from the 2024 cohort and post-intervention data from the 2023 cohort (pre-intervention data were not collected until the 2024 cohort). As a quality improvement project, families were not required to complete surveys to participate in Team Survivors, and no identifiable information was collected. Families were informed that data collection was intended to identify strengths and areas of growth for the program to improve participation and outcomes for future cohorts. Due to low post-intervention response from survivors (*n* = 2), only caregiver data were included in the pre–post analysis. Pre–post analyses were supplemented by descriptive and narrative analyses of qualitative data from survivors and caregivers in the 2023 and 2024 cohort about their reason for participating, experience, and overall satisfaction, to provide a holistic perspective on the program. 

Surveys were conducted through Qualtrics and distributed to caregivers and survivors through a QR code. The online surveys included qualitative and quantitative measures assessing survivors’ HRQoL, exercise self-efficacy, and satisfaction and perception of Team Survivors before and after participating. The QR codes were disseminated to caregivers and survivors in the 2024 cohort at the information session before the first training session and at the end-of-the-year celebration. The survey was only disseminated during the end-of-the-year celebration for the 2023 cohort. A program coordinator (JM) also sent the QR codes via email at the same timepoints to bolster participation. Caregivers and survivors were administered the same measures with the wording adapted based on their role (e.g., “What are your hopes for your child participating in Team Survivors?” for caregivers and “What are your hopes for participating in Team Survivors?” for survivors). 

### 2.2. Participants 

Only patients and families that participated in Team Survivors were invited to complete the surveys. To be eligible to participate in Team Survivors, survivors need to be between 9 and 17 years old on race day, have previously received a HSCT or treatment for pediatric cancer, be in maintenance therapy or off treatment, and be a patient at Children’s Wisconsin MACC Fund Center for Cancer and Blood Disorders. Additionally, each survivor’s primary oncologist or transplant physician was required to provide medical approval for participation. Exclusion criteria for Team Survivors include being in active treatment for cancer or HSCT, being under the age of 9 years old or over the age of 17 years old on race day, being unable to commit to attending the training sessions, and not receiving medical approval from their primary physician. As a quality improvement project focused on improving local practice and not intended to generate generalizable knowledge, ethical review and approval were waived for this study.

Recruitment is conducted via fliers posted in the clinic and mailed to eligible patients, the MACC Fund Center Facebook page, staff referrals, and follow-up phone calls to families who have previously indicated interest. The program coordinator (JM) determines eligibility for each survivor after consulting with medical and psychosocial providers. Cohorts are limited to 12 survivors a year due to funding and bandwidth. The 2023 cohort had 9 participating survivors, and the 2024 cohort had 11 participating survivors. 

### 2.3. Materials 

#### 2.3.1. Exercise Self-Efficacy

Exercise Self-Efficacy Scale (ESES) [42]: The ESES consists of 10 items designed to assess confidence in one’s ability to engage in physical activity and exercise. Items are not disease-specific and assess general exercise self-efficacy (e.g., “I am confident I can overcome barriers and challenges with regard to physical activity and exercise if I try hard enough”). Items are rated on a 4-point Likert scale ranging from 1 (not at all true) to 4 (always true). Total scores range from 10 to 40, with higher scores indicating greater perceived exercise self-efficacy. The ESES has high internal consistency (*a* = 0.87–0.93), adequate retest reliability (*ICC* = 0.79–0.81), and moderate construct validity [42,43]. Given the generic nature of the measures, it has been employed in research for a wide range of medical populations across the developmental spectrum [44,45,46]. Wording was modified for caregivers to reflect that this is their report of their child’s self-efficacy. For example, I am confident **I** can be physically active or exercise even when **I am** tired was changed to, I am confident **my child** can be physically active or exercise even when **they are** tired.

#### 2.3.2. Health-Related Quality of Life 

Pediatric Quality of Life Inventory (PedsQL) Generic Core Scale [47]: Parallel parent-proxy and child self-report versions of the PedsQL were used to assess overall quality of life across the following four functioning domains: physical (8 items), emotional (5 items), social (5 items), and school (5 items). The PedsQL items are represented as reversed scores and are linearly transformed to a 0–100 scale with higher scores indicating better HRQoL. Subscale scores for each domain are calculated by summing the items within the domain and dividing them by the number of items. The total score is similarly computed by summing all items and dividing them by the total number of items. Psychosocial and Physical Health Summary Scores are created from the subscales. The Psychosocial Health Summary Score is computed by combining the emotional, social, and school functioning subscales and dividing by the number of items included. The Physical Health Summary Score is calculated using the same items as the physical subscale. The PedsQL has repeatedly shown good internal consistency, construct validity, and sensitivity to changes in disease severity and treatment responsiveness [47,48,49]. 

#### 2.3.3. Feedback on Participation and Program Satisfaction

Caregivers and survivors answered additional qualitative and quantitative items to elicit feedback on their reason for participating, experience during the program, perceived benefits, and overall satisfaction with Team Survivors. Items were structured as dichotomous questions, 5-point scale questions, or free-response questions.

### 2.4. Intervention 

Team Survivors is a 12-week program adapted from Team Phoenix [50], an all-women adult breast cancer survivor triathlon team initiated at Milwaukee’s Aurora Research Institute in 2011. Pediatric survivors attend twice-weekly group training sessions of swimming, biking, and running in preparation for completing a community triathlon at the end of the program. Survivors are provided with a bike, helmet, bike light, goggles, swimsuit, backpack, team jersey, and custom-fit running shoes. All equipment is individualized, with adaptive equipment provided based on survivors’ physical abilities. Training sessions are led by a dedicated athletic trainer (KO) from the Department of Sports Medicine, a secondary athletic trainer who is available to help with medical coverage, and 3–5 volunteers from the hospital or community who provide additional support and one-on-one instruction. Survivors do not need to have previous experience in swimming, biking, or running. One-on-one support is provided during training sessions based on each survivor’s ability level, and the sessions are individualized to ensure success and enjoyment for each survivor. Each session consists of a warm-up, workout, team games, and a cool-down. Team games are designed to build friendships and comradery while simultaneously developing skills and endurance. At the end of each training session, the team reviews their training plan to set goals for the next week. A dietician and sports psychologist each attend various training sessions to discuss other factors impacting sports performance, including the appropriate nutrition for training sessions and race day, performance anxiety, and coping strategies. Alumni of Team Survivors are invited back to training sessions to stay active, build community, and create informal mentoring opportunities. At the end of the program, survivors compete in a community triathlon with healthy, same-aged peers and engage in an end-of-year gathering to celebrate and reflect on the team’s accomplishments. The program is philanthropically funded.

### 2.5. Data Analysis 

All statistical analyses were conducted using IBM SPSS Statistics (Version 25). Two-tailed repeated measures *t*-tests were used to compare pre-intervention and post-intervention caregiver-reported scores on the ESES and PedsQL for the 2024 cohort. Descriptive and narrative data on reasons for participating, perceived experience, and overall satisfaction from caregivers and survivors of the 2023 and 2024 cohorts are included to provide greater detail of the perceived effects of participation. Caregiver and survivor feedback is presented as a narrative summary, given the small sample size and similar themes reported across items.

## 3. Results

### 3.1. Demographics 

The 2023 cohort included nine survivors, with five males (56%), three females (33%), and one survivor who identified as non-binary (11%). Ages ranged from 8 to 13 years with an average age of 9.89 years old (*SD* = 1.68). Average time since treatment completion was 4.95 years (*SD* = 2.99; 7 months to 9 years). Leukemia was the most common diagnosis for this cohort (56%). Other diagnoses included Wilms tumor (11%), Hodgkin lymphoma (11%), neuroblastoma (11%), and anaplastic ependymoma (11%). Demographics of the caregivers were not collected for either cohort. 

The 2024 cohort consisted of 11 survivors, with 8 males (73%) and 3 females (27%) ranging in age from 8 to 15 years old (*M* = 11.50, *SD* = 2.16). Six survivors received treatment for leukemia (ALL; 55%), four survivors for Hodgkin lymphoma (36%), and one survivor, respectively, for retinoblastoma (1%), medulloblastoma (1%), and sickle cell disease (1%). Two survivors received a HSCT as part of their treatment. Time since treatment completion ranged from 1 month to 9 years with an average of 3.46 years (*SD* = 2.91 years). 

As a quality improvement project, survivors and caregivers were not required to complete surveys to participate in Team Survivors, and no identifiable information was collected to determine who did and did not complete the surveys. Survey response rates can be found in Table 1. Due to low response from survivors, only caregiver data are included in the analyses.

### 3.2. Pre- and Post-Intervention Outcomes 

Descriptive data of the 2023 cohort post-intervention and 2024 cohort pre- and post-intervention surveys can be found in Table 2. Inferential statistics for the pre–post *t*-test of the 2024 cohort are presented in Table 3. Pre- and post-intervention scores did not significantly differ on the ESES or any of the PedsQL scales. The average ESES scores differed by less than one point between the 2023 post-intervention scores and 2024 pre- and post-intervention scores. The 2024 post-intervention scores improved from pre-intervention on all PedsQL domains by an average of 3–5 points. The 2023 post-intervention scores were notably higher than both the 2024 pre- and post-intervention scores.

### 3.3. Feedback on Participation and Program Satisfaction

All survivors (100%) in both cohorts completed the triathlon, and 96% of survivors and caregivers reported feeling prepared for the triathlon. More than half of the families (57%) expressed interest in competing in another triathlon, with the majority (91%) stating they planned to continue engaging in running, biking, or swimming after completing Team Survivors. When asked to rate their satisfaction on a 5-point Likert scale (1 = very unsatisfied; 5 = very satisfied), families, on average, reported being “satisfied” or “very satisfied” with the coaching staff (*M* = 5.00), program coordination and logistics (*M* = 5.00), practice locations (*M* = 4.74, *SD* = 0.62), and practice times (*M* = 4.83, *SD* = 0.50). 

Prior to starting the program, families were asked what they hoped to gain from participating, as well as any worries or concerns they had. Caregivers were primarily interested in improving their child’s physical activity and self-confidence, citing hopes their child would build confidence and stamina, lose weight, and gain back muscle tone and strength (77%). Survivors reported hoping to develop new friendships and “have fun” (71%) as their primary reasons for participating. Both caregivers and survivors expressed concerns about anxiety, managing pain, possible injury, and low motivation and endurance before starting the program.

After completing the program, caregivers and survivors were asked if they felt they benefitted from participating in Team Survivors and how it impacted the survivors’ physical activity and exercise. Caregivers and survivors unanimously reported benefiting from Team Survivors. Families reported increased motivation for and engagement in physical activity as well as improved confidence and endurance (83%). One caregiver reported their child “can be difficult to motivate, but [Team Survivors] was exciting for him and one of the few times he’s been happy to work hard toward a goal. I was so happy to see him work towards something and have success in the end”. Several caregivers highlighted how Team Survivors helped their child cope with challenges related to physical activity and modify beliefs about recovery. One caregiver stated their child “often feels winded or gets a belly ache with too much activity. He didn’t seem to complain during or after practice and he had fun doing it! It was great to push him under the care and guidance of experienced medical professionals and survivors”. This sentiment was similarly reflected in athletes’ responses, with one reporting that Team Survivors helped them pace themselves while training and another stating they felt more “confident and comfortable” with different forms of physical activity. 

Increased social engagement was also highlighted by several caregivers and survivors (57%). Families observed survivors developing friendships and strengthening important social skills as they progressed through the program. Some families added that participating with other pediatric survivors created a positive environment where their child felt more comfortable engaging in physical activity. For example, one caregiver stated their child benefited from training with “other kids at her same level, needing help with bik[ing] and swim[ming] so she wasn’t the only one”. The impact on social connectedness was not limited to peer relationships, as some caregivers shared that they started engaging in physical activity with their child as a result of their participation in Team Survivors. One caregiver described buying a bike for themself after Team Survivors so parent and child could go on bike ride together.

Families also discussed how participating in Team Survivors helped provide a feeling of normalcy with other children that had been lacking since their diagnosis and treatment (26%). One caregiver described Team Survivors as “a wonderful opportunity to be normal but accommodated”. Emphasis on feeling normal was highlighted when families were asked about competing in a triathlon with other children without chronic medical conditions. One caregiver reported, “it reminded my son how special he is and how blessed he is to be alive and able to train. For the most part though, he just felt like a normal kid training with other normal kids”. Overall, caregivers and survivors had little feedback for ways to improve Team Survivors beyond suggesting a social gathering at the start of the program for survivors and families to meet. Families expressed gratitude for the program to further support survivors as they transition back to post-treatment life. 

## 4. Discussion

Inadequate physical activity is an ongoing issue for pediatric survivors of childhood cancer and HSCT, increasing the risk for chronic health conditions and decreasing quality of life in survivorship [51,52]. Improving physical activity for this population is a key goal to address immediate and long-term treatment effects. However, inadequate physical activity in pediatric cancer and HSCT survivors is not solely attributed to the physical effects of treatment. Barriers to physical activity for this population are multifaceted and include low motivation, health-related fears, and anxiety; limited self-efficacy; treatment-related physical limitations and side effects; caregiver concerns and perceived self-efficacy; and limited opportunities for physical activity [18,53,54]. Thus, improving physical activity for survivors of childhood cancer and HSCT likely requires a multifaceted intervention that targets the different determinants at play.

Team Survivors is an innovative program that addresses common physical and psychosocial challenges experienced by pediatric cancer and HSCT survivors. In addition to improving survivors’ physical activity through adaptive triathlon training, the program is structured to establish comradery with other survivors and the wider community, to enhance motivation and self-efficacy, and to reinforce adaptive coping and beliefs about physical activity. The preliminary data presented here support the feasibility and acceptability of Team Survivors in improving physical activity and health-related quality of life for pediatric survivors. The lack of significant findings for the quantitative analysis is unsurprising given the limited sample size; however, the overall benefits depicted by the mixed method approach suggests caregivers and survivors perceived participation in Team Survivors to positively impact survivors’ physical activity and more broadly improve psychosocial functioning. 

The improved social engagement and connectedness reported by caregivers and survivors was further indicated by the overall improvements in caregiver-reported HRQoL. While differences in the pre–post analysis of HRQoL did not reach statistical significance, the 3–5 point gains across domains may reflect clinically significant gains in daily quality of life and functioning reflected in qualitative data. When applying PedsQL cut-off scores to differentiate by condition severity, the 2024 cohort of survivors’ baseline HRQoL aligned with children with moderate-to-major chronic health conditions; their scores after completing Team Survivors were more similar to children with moderate-to-mild chronic health conditions [55]. Notable, 2023 cohort’s caregiver-reported HRQoL better aligned with healthy children than those of pediatric cancer survivors [48] or of other children with chronic medical illnesses [55]. Without pre-intervention data for the 2023 cohort, it is unclear if this cohort made greater gains between pre- and post-participation than the 2024 cohort or if their HRQoL was already higher at baseline. Caregivers’ and survivors’ reports of improved social engagement with peers and family, increased feelings of normalcy, and strengthened coping abilities help clarify how Team Survivors may enhance HRQoL. 

Feeling “normal” was a particularly poignant benefit of Team Survivors, as reported by caregivers and survivors. Children are routinely isolated from peers while undergoing HCST and cancer treatment and often experience debilitating side effects from treatment that can make it challenging to resume previous activities when treatment is over [56]. They also frequently miss developmentally expected milestones and activities, such as regularly attending school, while undergoing physical and psychosocial stressors that most of their peers do not experience [57]. Team Survivors helps combat isolation and disconnection by providing an opportunity to engage in several of the experiences they missed due to treatment with other survivors and the wider community. The psychosocial improvements that survivors gain from Team Survivors may also have a reciprocal effect on their physical activity. Developing a sense of community through group exercise has been shown to improve exercise adherence and impact perceptions of physical exertion and enjoyment [58,59]. For example, experimental studies by Davis et al. [60] found synchrony in exercise among athletes can act as a cue for social support, which can reduce perceptions of fatigue and pain. If Team Survivors successfully enhances social connectedness and positively affects the perception of physical activity, survivors may be more motivated to continue regular physical activity after completing the program as a way to maintain those social relationships and improved quality of life.

Interest in creating physical activity interventions for childhood cancer survivors is rapidly growing [52,61,62], allowing important mechanisms related to improved physical activity and quality of life to emerge. Meta-analyses of these interventions suggest that supervised, multimodal, group-based programs, like Team Survivors, produce the most promising outcomes [63,64,65]. Unsurprisingly, supervised interventions have greater retention and adherence, as well as increased levels of daily physical activity and improved fatigue than unsupervised interventions, such as web-based or distance-based programs [64]. Furthermore, more comprehensive programs that utilize a group format and incorporate educational and psychosocial components have been the most effective in improving quality of life [63,65]. In a feasibility study of FitSurvivor, a group-based exercise intervention with a complementary phone app, participants had greater engagement with the group-based intervention phone app [66]. When asked what they found most beneficial, participants reported similar themes to those identified for Team Survivor, including improved encouragement, motivation, education, and social enjoyment. Both the qualitative and quantitative data presented here support the importance of a supervised, multimodal, and group-based approach to address several of the barriers to physical activity pediatric survivors face, including limited peer support, limited motivation and self-confidence, and limited knowledge and misconceptions about physical activity [17,67]. The elements unique to Team Survivor, such as establishing a shared goal and re-integration into the community, warrant further assessment to determine if they help improve effectiveness and address barriers.

The improvements in physical functioning from the qualitative data were not reflected in the quantitative analysis of the ESES. There are several possible explanations why there was little change in caregiver-reported exercise self-efficacy on the ESES, despite families overwhelmingly reporting improved survivors’ physical activity, endurance, and self-confidence. While the ESES was chosen because it assesses general self-efficacy, it may lack the sensitivity needed to effectively detect changes in this population. A disease-specific measure with items specifically assessing physical activity within the context of HSCT and cancer may better capture the impact of treatment on survivors’ self-efficacy for physical activity. For example, having items about treatment-related deconditioning and common late effects of treatment, such as cardiovascular disease or pain, may elicit a more nuanced presentation of physical activity in survivorship. Caregiver-reports of exercise self-efficacy may also have been impacted by recency bias. If survivors were not regularly engaging in physical activity post-treatment, caregivers may not have witnessed their child struggle with exercise. Thus, caregivers may have reported feeling confident their child could be physically active even when they are tired, as is asked on the ESES, not because they have seen it but because they have not had enough opportunities to see their child trying to be physically active when tired. 

There are several limitations to this study that should be considered when interpreting these findings. As previously mentioned, the study was not adequately powered to detect significant effects, if they were present, due to the small sample size. While changes in descriptive HRQoL data between pre–post timepoints were encouraging, a larger sample is needed to demonstrate whether Team Survivors can meaningfully impact survivors’ physical and psychosocial functioning. As previously discussed, cohorts are limited to 12 survivors a year due to funding and bandwidth, which also limit the sample size. We will likely need to rely on cross-sectional data to adequately assess the program’s impact. Secondly, there was no control group to determine whether changes in physical activity and psychosocial functioning can be attributed to Team Survivors as opposed to other factors. Thirdly, we did not include objective measures of physical health (e.g., VO2 max) that would have allowed us to better assess the direct effect Teams Survivors had on different facets of health. A quantitative measure of physical exercise was initially included in data collection but was discarded due to user error. Further iterations of this program would benefit from adding a more user-friendly quantitative measure to better capture the direct impact on physical activity, as opposed to relying on ESES and PedsQL as proxies. Finally, we were only able to conduct quantitative analyses on caregivers’ responses due to the low response rate from survivors. Both caregiver and survivors’ perceptions are needed to determine the success of Team Survivors as motivation from both is necessary for ongoing physical activity after the program. Follow-up studies may consider ways of improving survivors’ response rate so they can be included in quantitative analyses. 

## 5. Conclusions

There are many factors that contribute to reduced physical activity in pediatric cancer survivorship, including deconditioning related to decreased activity while on therapy, fatigue as a late effect of treatment, and reduced confidence in one’s ability to engage in physical activity. Team Survivors offers preliminary support as a multifaceted intervention program to promote survivors’ self-confidence, endurance, and motivation for physical activity. The focus on enhancing social connectedness and re-integrating survivors back into their community is unique to the structure of Team Survivors and may reinforce ongoing physical and psychosocial gains long after survivors complete the program. Additional studies and a larger sample size are needed to further delineate the benefits of the program. 

## Figures and Tables

**Table 1:** Response rate to the quality improvement survey by responder type and timepoint.

Responder	2023 Post-Intervention Response Rate **	2024 Pre-Intervention Response Rate	2024 Post-Intervention Response Rate
Survivors	2	7	2
Caregivers	10 *	14 *	11 *
Total	12	21	13

* Totals are greater than the number of survivors who competed in Team Survivors as more than 1 caregiver was allowed to complete the survey for the same survivor. ** Pre-intervention surveys were not collected for the 2023 cohort.

**Table 2:** Descriptive data of caregiver-reported measures.

	2023 Cohort Caregivers **	2024 Cohort Caregivers	
	Post-Intervention (*n* = 10) *M (SD)*	Pre-Intervention (*n* = 14) *M (SD)*	Post-Intervention (*n* = 11) *M (SD)*
ESES ^1^	33.5 (6.4)	33.7 (3.9)	33.0 (3.8)
PedsQL Total Score ^2^	81.6 (10.6)	72.0 (15.9)	76.1 (10.7)
PedsQL Psychosocial ^3^	81.0 (12.3)	71.6 (16.7)	75.8 (10.8)
PedsQL EF ^4^	78.4 (15.6)	68.6 (19.5)	74.1 (14.6)
PedsQL SF ^5^	83.0 (13.4)	76.8 (17.8)	80.5 (16.8)
PedsQL SchF ^6^	81.5 (14.7)	69.3 (20.1)	72.7 (16.3)
PedsQL Physical ^7^	83.4 (7.7)	73.4 (16.3)	77.0 (12.8)

^1^ Exercise Self-Efficacy Scale; ^2^ Pediatric Quality of Life Inventory Total Score; ^3^ Pediatric Quality of Life Inventory Psychosocial Functioning; ^4^ Pediatric Quality of Life Inventory Emotional Functioning; ^5^ Pediatric Quality of Life Inventory Social Functioning; ^6^ Pediatric Quality of Life Inventory School functioning; ^7^ Pediatric Quality of Life Inventory Physical Functioning. ** Pre-intervention surveys were not collected for the 2023 cohort.

**Table 3:** Inferential statistics of caregiver-reported scores pre- and post-intervention from the 2024 cohort (*n* = 11).

Measures/Scales	*t*	*p*	*d*
ESES ^1^	0.4	0.7	3.9
PedsQL Total Score ^2^	−0.7	0.5	12.9
PedsQL Psychosocial ^3^	−0.7	0.5	14.4
PedsQL EF ^4^	−0.8	0.4	17.5
PedsQL SF ^5^	−0.5	0.6	17.5
PedsQL SchF ^6^	−0.5	0.7	18.6
PedsQL Physical ^7^	−0.6	0.6	14.9

^1^ Exercise Self-Efficacy Scale; ^2^ Pediatric Quality of Life Inventory Total Score; ^3^ Pediatric Quality of Life Inventory Psychosocial Functioning; ^4^ Pediatric Quality of Life Inventory Emotional Functioning; ^5^ Pediatric Quality of Life Inventory Social Functioning; ^6^ Pediatric Quality of Life Inventory School functioning; ^7^ Pediatric Quality of Life Inventory Physical Functioning.

## Data Availability

The data presented in this study are available upon request from the corresponding author. The data are not publicly available due to privacy reasons.
